# Olfactory Identification Improve the Prediction of Episodic Memory Function in Individuals at Risk of Alzheimer's Disease: Results from the CIMA‐Q Cohort

**DOI:** 10.1002/alz70857_097899

**Published:** 2025-12-24

**Authors:** Benoît Jobin, Johannes Frasnelli, Natalie A. Phillips, Benjamin Boller, CIMA‐Q Group

**Affiliations:** ^1^ Research Center of the Hôpital du Sacré‐Cœur de Montréal, Montréal, QC, Canada; ^2^ Université du Québec à Trois‐Rivières, Trois‐Rivières, QC, Canada; ^3^ Centre de recherche de l'Institut universitaire de gériatrie de Montréal, Montréal, QC, Canada; ^4^ Concordia University, Montréal, QC, Canada; ^5^ Institut Universitaire de Gériatrie de Montréal, Montréal, QC, Canada; ^6^ Consortium pour l'Identification précoce de la Maladie d'Alzheimer ‐ Québec, Montréal/Québec/Sherbrooke, QC, Canada

## Abstract

**Background:**

Olfactory identification is impaired early in the clinical continuum of Alzheimer's disease (AD) and is already observable in mild cognitive impairment (MCI). A recent meta‐analysis showed significant associations between olfactory identification and episodic memory scores in older adults without cognitive impairment. Thus, olfactory identification could potentially serve as a marker for episodic memory decline in individuals at risk of AD. This study aimed to (1) evaluate the predictive value of olfactory identification on episodic memory functioning in individuals with MCI and subjective cognitive decline (SCD) and (2) explore the role of olfactory identification in discriminating between the two groups.

**Method:**

Using the University of Pennsylvania Smell Identification Test (UPSIT), we assessed the olfactory identification function of 93 participants: 48 with SCD (mean age: 75.82, SD: 5.64) and 45 with MCI (mean age: 80.08, SD: 5.86) from the Consortium for the Early Identification of Alzheimer's Disease (CIMA‐Q) cohort. Episodic memory was evaluated using immediate and delayed recall scores from the Rey Auditory Verbal Learning Test (RAVLT). LASSO regression models were applied, with 80% of the data used for training and 20% for testing. Linear Discriminant Analysis (LDA) was applied to assess group classification accuracy using the UPSIT score.

**Result:**

The UPSIT score demonstrates significant associations with both immediate (*ß* = 0.56, *p* < .001) and delayed recall scores (*ß* = 0.19, *p* < .001). Incorporating the UPSIT score in predictive models ‐ along with age, sex, and education ‐ improved the explained variance for RAVLT immediate recall from 9% to 19% and for delayed recall from 8% to 20%. The MCI group exhibited significantly lower UPSIT scores compared to the SCD group (*p* = .01); LDA achieved moderate accuracy (69%) for distinguishing groups, with higher specificity to rule out MCI (79%) than sensitivity to detect it (58%).

**Conclusion:**

Olfactory identification enhanced the prediction of episodic memory in individuals with SCD or MCI; highlighting its potential utility as a screening tool for cognitive decline associated with AD. However, because olfactory impairment is not specific to AD, further research is necessary to elucidate the underlying mechanisms driving this impairment.

